# The Importance of Establishing a Technic as Well as Literary Standard for College Entrance

**Published:** 1898-11

**Authors:** S. H. Guilford

**Affiliations:** Philadelphia, Pa.


### THE DENTAL REGISTER.

VOL. LII.] NOVEMBER, 1898. [No. 11.

### COMMUNICATIONS.

### The Importance of Establishing a Technic as well as Literary Standard for College Entrance.

BY S H. GUILFORD, D.D S., PH D., PHILADELPHIA, PA.

Read before the National Association of Dental Faculties, August 29, 1898.

The good results accomplished by this Association in its sixteen years of existence are not only universally conceded, but will ever remain as proof of the wisdom of its organization and its general endeavors to elevate the standard of practice. It was realized that this could only be done by sending into the profession men better equipped for practice than the majority of those then entering it. This required certain changes in the prevailing methods of college instruction, some of which have gradually been brought about while others are in course of development. The Association first addressed itself to lengthening the course of study. The recognition of five years' practice as an equivalent of one year's course in college was done away with, and an invariable two years' course demanded.

Then came the lengthening of the winter term from four to five and subsequently to six months, after which another year was added to the college curriculum.

With all these advancements, however beneficial as they were in their results, it was found that a proportion of the yearly graduates were not up to the standard demanded either by the public or the profession, and another change became necessary to remedy the condition. At the time of the organization of this Association, and for many years afterward, there were very few dental colleges that inquired into the earlier educational training of those who applied for admission. It seemed to have been taken for granted that any one applying for admission must have had sufficient mental training to enable him to grasp and pursue the various studies of the curriculum. This proved to be erroneous, for it was found that while a student might, by close application, manage to pass his examination in the theoretical branches, he did not have that general grasp of these subjects which was necessary to make him a well-rounded man.

Following this discovery came the adoption of entrance requirements, which necessitated a certain amount of mental training in the schools before the candidate could be allowed to begin his collegiate studies. These requirements were very moderate at first, but were gradually increased until they equaled the completion of a full grammar course.

This far the plan had worked admirably, principally because it was gradual. Two years ago, however, a further advance was decided upon by which in the course of a few years the entrance requirements were to equal completion of a high school course. This change was so radical in character that it worked a hardship upon many students who were not able to meet it, and who in consequence were debarred from college entrance.

As a result of this, one year ago the latest advance was annulled and the requirements reduced to their previous standard. This retreat from an advanced position was regretted by many schools, and the question of some advancement from our present standard will doubtless come before the Association at its present meeting.

In anticipation of this, your essayist decided to prepare this paper for the purpose of presenting certain views upon the subject and offering them for your consideration.

As previously mentioned, the raising of the entrance requirements to equal a completed grammar school course has proven itself a wise act, and none have cause to regret it. With less preparatory training it was found that the student's mental faculties had not been properly awakened nor correct habits of study formed, and that he was in consequence placed at a disadvantage in trying to acquire a knowledge of at least some of the more abstruse subjects which he was expected to master.

In view of the fact that the advancement to the present standard has worked well, the question naturally arises as to whether a further advancement would not be advisable, and, if so, what form it should take? Strange to say, we have thus far been viewing and treating the subject of preliminary requirements from a single standpoint. All of our discussions, as well as our enactments, have dealt solely with the mental acquirements and possibilities of the proposed student, entirely overlooking or ignoring the equal or more important feature of manual dexterity or mechanical bent.

All of us are fully aware of the absolute importance of mechanical talent in the practice of our profession, and we are equally cognizant of the fact that unless this talent is innate it will always be lacking, for it cannot be acquired. No amount of training and instruction can develop a skillful mechanic out of one who lacks the mechanical instinct. If this be so, is it not important that we, as teachers, see that those who place themselves under our care for preparation for their life-work are possessed of this necessary qualification? In former times, before the wave of progress had swept across the beaten path of dental education, when the student received his preliminary, and at times the greater part of his dental, training in a preceptor's office, or rather laboratory, it was an almost universal custom for the practitioner, before accepting a student, to ascertain whether he possessed a natural bent in the line of mechanics. This was done by inquiring into the young man's turn of mind, his fondness for tools and their employment in construcing some of the simple mechanisms so necessary to the complete happiness of boyhood. In addition to this it was customary to accept the student for a certain period upon probation, to still further ascertain his adaptability to his proposed life-work.

While in these latter times we recognize the shortcomings of our predecessors in not demanding at least some educational requirements from their students, may we not at the same time take a hint from their methods, and incorporate some of their requirements into our own? In other words, has the time not fully arrived when we should demand mechanical talent as well as scholastic acquirements as preliminaries to entrance upon the study of dentistry?

It would seem that in this matter, as in many others, we have been rather blindly following in the footsteps of our sister profession, medicine, not fully appreciating the differences that exist between them. Dentistry occupies rather a unique position among the sciences and professions, in that to be of the greatest service to mankind the practitioner must necessarily be possessed of considerable manual dexterity.

This is not required of the lawyer, the theologian, or the physician in ordinary practice, for their success depends mainly, if not entirely, upon the development and use of their mental faculties. For one undertaking the study of any of these professions it is, therefore, quite proper that the only qualification demanded should be a scholastic one.

Should the student of medicine prove to be possessed of mechanical talent, he will, after graduation, naturally drift into the special practice of surgery, which will be more to his taste, and afford him a better field for the employment of manipulative skill. Should his taste not run in the mechanical line, he still has in the domain of general practice, and some of the specialties, a large field for successful effort.

With us it is different. To properly serve the needs of his patients the dentist must be skillful with tools, for so large a part of his daily work is manipulative in character. If he lacks this skill he must prove a failure, for in the practice of dentistry there is no place for the employment of the mental faculties alone as there is in medicine.

The vocation of the instrumental musician bears some little resemblance to our own, in that it requires for its successful pursuit, not only the development of the mental and esthetic qualities, but an absolute dependence upon manipulative ability. Without the latter the former quality would be of no avail. A teacher of instrumental music would probably prefer to have as his student one with a liberal education, for he would add luster to his chosen calling; but he would certainly not accept or retain as a pupil, no matter what his literary attainments may be, one who was lacking in technical ability or possibility.

Why, therefore, should we do less?

The dentistry of to-day owes much of its progress and high standing to the class of men who entered it from thirty to sixty years ago under the private studentship system. Almost without an exception they were men possessed of a high order of mechanical and inventive ability, and they were so because they were selected from the mass by their preceptors.

It, therefore, seems to me that it would only be the part of wisdom for us to so amend our requirements as to include manipulative ability, and where this is lacking to reject the student and advise him to take up some other calling. It certainly does not seem just to accept a student who is by nature lacking in that quality which is absolutely essential to his success in practice.

With our greatly improved methods of systematic technic instruction we have certainly accomplished good results with the material given us, but how much better might have been the results with the material properly culled? Many students, as we all know, manage to work along through college, performing their allotted tasks and passing the required examinations, who, we are morally certain, will not be successful in practice because all that they accomplished was performed in a labored way, without any display of actual skill.

Are we just to them and to the public in permitting this? Should we not discover the lacking quality before accepting them, or find some way of discovering it in the early part of their course and kindly advise them to change their vocation?

If by some extra effort on our part we were able to develop skill where natural ability is lacking the conditions would be different, and we would be relieved from the necessity of considering the question; but, unfortunately, we cannot grow the plant where seed or soil is lacking.

The question now arises, What shall the mechanical standard be, and how may it best be incorporated with the other requirements? This is not for me to answer. It is a problem, and its solution will require the united wisdom of the members of this Association.

By way of suggestion, however, I would offer the following:

1. The student should be assigned a desk or bench in the laboratory, furnished with the necessary tools and material, and be given an appliance or device which he is to reproduce as accurately as possible.

2. The task assigned should be such as to preclude the probability of his having done work of exactly similar character before, so as to guard against mere automatic repetition.

3. The ordinary laboratory processes, involving no special skill, such as repairs or additions to vulcanite plates, should be excluded.

4. In cases where the candidate has had no experience in the use of some of our special tools or processes, such as soldering, swaging, etc., the test should be simple in character, and might consist in requiring him to reproduce from a block of wood, by means of saw, file, and penknife, some geometrical form, as a cube, pyramid, or rhomb.

5. In cases where the applicant has had some laboratory instruction or practice before coming to college, the test should be a little more severe in character. Inasmuch as regulating appliances are so varied in character, and often combine a number of different manipulations in their construction, such as filing, bending, soldering, etc., the construction of one of unusual design would probably furnish the best all-around test of ability.

6. During the test the student should be isolated until the task is completed. A competent demonstrator should watch the progress of the work from time to time, so as to form an opinion of the candidate's handiness with tools, but should offer no aid, even in the way of suggestion.

